# Picking up a Fight: Fine Tuning Mitochondrial Innate Immune Defenses Against RNA Viruses

**DOI:** 10.3389/fmicb.2020.01990

**Published:** 2020-08-31

**Authors:** Sourav Dutta, Nilanjana Das, Piyali Mukherjee

**Affiliations:** School of Biotechnology, Presidency University, Kolkata, India

**Keywords:** mitochondria, innate immunity, mitochondrial antiviral signaling, retinoic acid inducible gene-I, RNA virus, cytokine storm

## Abstract

As the world faces the challenge of the COVID-19 pandemic, it has become an urgent need of the hour to understand how our immune system sense and respond to RNA viruses that are often life-threatening. While most vaccine strategies for these viruses are developed around a programmed antibody response, relatively less attention is paid to our innate immune defenses that can determine the outcome of a viral infection *via* the production of antiviral cytokines like Type I Interferons. However, it is becoming increasingly evident that the “cytokine storm” induced by aberrant activation of the innate immune response against a viral pathogen may sometimes offer replicative advantage to the virus thus promoting disease pathogenesis. Thus, it is important to fine tune the responses of the innate immune network that can be achieved *via* a deeper insight into the candidate molecules involved. Several pattern recognition receptors (PRRs) like the Toll like receptors (TLRs), NOD-like receptors (NLRs), and the retinoic acid inducible gene-I (RIG-I) like receptors (RLRs) recognize cytosolic RNA viruses and mount an antiviral immune response. RLRs recognize invasive viral RNA produced during infection and mediate the induction of Type I Interferons *via* the mitochondrial antiviral signaling (MAVS) molecule. It is an intriguing fact that the mitochondrion, one of the cell’s most vital organelle, has evolved to be a central hub in this antiviral defense. However, cytokine responses and interferon signaling *via* MAVS signalosome at the mitochondria must be tightly regulated to prevent overactivation of the immune responses. This review focuses on our current understanding of the innate immune sensing of the host mitochondria by the RLR-MAVS signalosome and its specificity against some of the emerging/re-emerging RNA viruses like Ebola, Zika, Influenza A virus (IAV), and severe acute respiratory syndrome-coronavirus (SARS-CoV) that may expand our understanding for novel pharmaceutical development.

## Introduction

Mitochondrion, also known as the “powerhouse” of the cell, is critically involved in cellular respiration and ATP synthesis. Apart from its canonical role in cellular energetics, it orchestrates cell fate through the process of apoptosis and mitophagy, thus maintaining cellular homeostasis (Tsujimoto and Shimizu, 2007; Murphy, 2009; Friedman and Nunnari, 2014; Mishra and Chan, 2014; Khan et al., 2015; Sliter et al., 2018). In recent years, several studies have pinpointed the crucial role of mitochondria in stimulating innate immune responses, as well as modulating parts of the adaptive immune response (Walker et al., 2014; Weinberg et al., 2015; Mills et al., 2017). The evolutionary conserved pattern recognition receptors (PRRs), expressed by most immune effector cells recognize conserved sequence within the pathogen and aids in their early detection and containment (Green et al., 2016). The Toll like receptors (TLRs) are a class of PRRs that recognize either dsRNA (TLR3) or ssRNA (TLR7/8) virus (Lester and Li, 2014; Hartmann, 2017; Miyake et al., 2018). The NOD-like receptor (NLR) family of PRRs is cytoplasmic receptors that form a multiprotein complex called “inflammasome” involved in the production of the pro-inflammatory cytokines IL1β and IL18 (He et al., 2016; Hughes and O’Neill, 2018). Another class of PRRs, the RIG-I like receptor (RLR) family involving retinoic acid inducible gene-I (RIG-I), melanoma differentiation-associated protein-5 (MDA-5), and laboratory of genetics and physiology 2 (LGP2) are cytoplasmic sensors of non-self and viral RNA (Vazquez and Horner, 2015; Sadler, 2017; Chow et al., 2018). A few of these receptors have been shown to augment mitochondria mediated antiviral innate immune responses *via* stimulation of Type I Interferon. Evolutionary conserved signaling intermediate in Toll (ECSIT) pathway, a component of the mitochondrial complex I, has been shown to enhance TLR7 responses *via* the mitochondrial adaptor protein tumor necrosis factor receptor (TNFR) associated factor 6 (TRAF6; Carneiro et al., 2018). NLRP3 has been shown to form the active inflammasome complex at the mitochondria by associating with the adaptor protein mitochondrial antiviral signaling protein (MAVS; Dorn, 2012; Haneklaus and O’Neill, 2015; Yabal et al., 2019). However, of special interest is the first identified RLR, RIG-I, which recognizes viral RNA that has a triphosphate moiety at its 5' end and has been shown to be targeted by some of the deadliest form of the RNA viruses (Kell and Gale, 2015; Dai et al., 2018). Following viral recognition, RIG-I binds to MAVS located on the outer surface of healthy intact mitochondria leading to interferon production and activation of the NFκB pathway (Kawai and Akira, 2007; Okamoto et al., 2018). This review speculates whether subversion of early viral sensing *via* the RIG-I/MAVS pathway could determine viral persistence within the host. Further, aberrant activation of the MAVS signalosome by the RLRs could cause hyperstimulation of the inflammatory responses and hence this arm of the innate immune defense could serve as a potential therapeutic target to combat highly communicable infectious RNA viruses.

The “Flu pandemic” over the last century has drawn particular attention to enveloped RNA viruses, a characteristic feature that empowers the virus with greater adaptability and high mutagenic potential, a key strategy in the evasion of host immune response and increased survivability within the host. Here, we systematically review our current understanding of the conserved host RIG-I/MAVS pathway and its regulation in some of the emerging/re-emerging RNA virus infections that include Ebola virus (EBOV) belonging to Filoviridae family, Zika virus (ZIKV) belonging to Flaviviridae family, Influenza A virus (IAV) belonging to Orthomyxoviridae family, and severe acute respiratory syndrome-coronavirus (SARS-CoV) belonging to Coronaviridae family. These viruses have been known to cause deadly outbreaks across the world and it is important to analyze whether key sensors of RNA viruses like the RIG-I/MAVS pathway are important targets of these viruses either to suppress of hyper-activate the immune responses.

## MAVS Signalosome in Enveloped RNA Virus

Mitochondria play an important role in antiviral immunity by eliciting and maintaining the RLR/MAVS signaling cascade. RLRs are soluble RNA helicase type receptors containing N-terminal tandem of caspase activation and recruitment domains (CARDs) and a DECH-helicase domain required for RNA binding and ATP hydrolysis (Kao et al., 2015; Brisse and Ly, 2019). All the three known RLRs (i.e., RIG-I, MDA-5, and LGP-2) are very efficient in distinguishing between cellular RNAs from those produced by RNA viruses (Züst et al., 2011). Upon recognition of viral RNA, one of the widely studied RLRs, RIG-I, binds to the downstream adaptor protein MAVS (also known as IPS-1, VISA, Cardif) at the mitochondria *via* CARD-CARD interaction (Liu et al., 2017). MAVS is an integral protein of the mitochondrial outer membrane that binds to the mitochondrial membrane *via* its C-terminal domain and acts as a key determinant of the antiviral signaling cascade (Xu et al., 2014). Following its interaction with RIG-I, MAVS bind with several kinases and other signaling molecules including TRAF3 and 6, TNFR associated death domain (TRADD), and TRAF associated NF-ĸB activator (TANK1) to form a large multimeric complex called the “MAVS signalosome” (Biacchesi et al., 2009; Vazquez and Horner, 2015). This structure ultimately leads to the activation of the interferon regulatory factor 3 (IRF3) and phosphorylation of IKKε to stimulate the NF-ĸB pathway leading to transcriptional activation of Type I Interferons and other inflammatory cytokines (Pothlichet et al., 2013; Refolo et al., 2020). Interferons in turn stimulate a plethora of interferon stimulated genes (ISGs) that aid in the containment of the viruses as well crosstalk with the adaptive immune response. Thus mitochondrial targeting *via* the MAVS signalosome by the viral proteins upon their entry appears to be a central executioner of antiviral responses as summarized in Table 1. In a continuous war with the host, viruses have evolved strategies to avoid MAVS mediated innate immune activation. For example, MAVS is expressed only on the surface of intact mitochondria and several studies suggest that RNA viruses alter mitochondrial metabolism and homeostasis that ultimately lead to mitochondrial damage and blocking interferon response *via* MAVS (Lei et al., 2009; Zhao et al., 2012; Wang et al., 2013; Choi et al., 2017; He et al., 2019). Over centuries, it has been found that enveloped RNA virus causes persistent human infections like the current COVID-19 pandemic (Schoeman and Fielding, 2019). Whether the viral envelope provides additional arsenal to the RNA viruses in the suppression of the protective interferon response *via* the MAVS signalosome is not known yet.

**Table 1:** Summary of viral proteins and their targets in mitochondria mediated antiviral response.

Virus	Viral proteins	Targets in mitochondrial functioning	References
Ebola virus	VP24	Inhibits RIGI pathway: binds karyopherin 𝛼1 and prevents localization of p-STAT in nucleus	(Reid et al., 2006; He et al., 2017)
	VP35	Inhibits RLR/MAVS signaling: binds PACT, binds dsRNA and prevents recognition by RIGI, inhibits IKK𝛆/TBK1 complex, inhibits TNF𝛼 mediated activation of PKR, causes SUMOylation of IRF7	(Cárdenas et al., 2006; Feng et al., 2007; Chang et al., 2009; Prins et al., 2009; Luthra et al., 2013)
Zika virus	NS4B	Induces mitochondrial elongation: inhibits activation of DRP1; disrupts MAVS signaling: inhibits phosphorylation of TBK1	(Keystone Symposia, n.d.; Wu et al., 2017)
	NS4A	Inhibits MAVS signaling: binds CARD domain of MAVS	(Ma et al., 2018)
	NS5	Restricts MAVS signaling: inhibits phosphorylation of IRF3 by binding TBK1, binds and degrades STAT2	(Grant et al., 2016; Lin et al., 2019)
	NS3	Binds and degrades MAVS	(Li et al., 2019)
Influenza A virus	PB2	Binds and inhibits MAVS	(Graef et al., 2010)
	PB1-F2	Binds and inhibits MAVS; induces mitophagy: interacts with TUFM and MAP1 LC3B/LC3B; disrupts MMP and induces apoptosis: binds VDAC1 and ANT	(Zamarin et al., 2005; Varga et al., 2012; Wang et al., 2020b)
	NS1	Inhibits RIG1 activation: degrades deubiquitylase OTUB1, binds TRIM25, binds CARD of RIG1	(Gack et al., 2009; Jahan et al., 2019; Jureka et al., 2020)
SARS-CoV	ORF3b	Translocates to mitochondria and inhibits RIG1/MAVS signaling; inhibits phosphorylation of IRF3	(Kopecky-Bromberg et al., 2007; Freundt et al., 2009)
	Nsp10	Induces ROS production: binds NADH 4 L subunit and cytochrome oxidase II; depolarizes inner mitochondrial membrane	(Li et al., 2005)

## Fine Tuning Interferon Responses at the Mitochondria

Following viral infection, our cellular defense machinery systematically induces a number of cytokines (both pro‐ and anti-inflammatory) that, in certain instances, may lead to hyperstimulation of the immune response in a positive feedback loop (Geoghegan et al., 2016; Shrivastava et al., 2016; Orzalli and Kagan, 2017). This leads to a catastrophic damage to the surrounding cells and the side effects of this manifests itself in some of the symptoms like fever, fatigue, nausea along with multiple organ failure (Chen et al., 2020; Hackbart et al., 2020). This has been observed not only in COVID-19 patients but also in case of other strains of the Flu virus, the MERS-CoV, and SARS-CoV1 leading to severe respiratory distress and increased mortality rates (DeDiego et al., 2014; Nieto-Torres et al., 2015; Liang et al., 2020). Hence, the question automatically arises is whether mitochondria can fine tune this response to prevent such overreaction of the immune cells.

**Figure 1:**
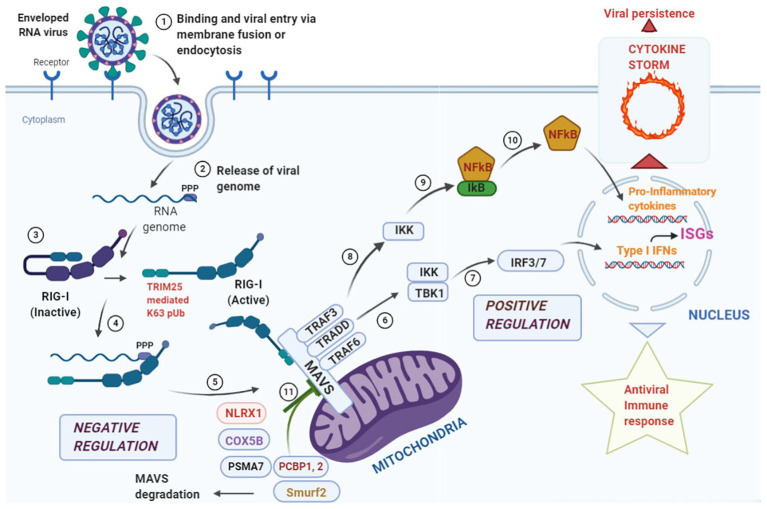
Overview of antiviral response at the mitochondrial antiviral signaling (MAVS) signalosome. Enveloped RNA virus can enter the cell *via* membrane fusion or endocytosis (Step 1). Following entry, the “ppp” group at the 5' end of the viral genome is recognized by the core domain of the retinoic acid inducible gene-I (RIG-I) like receptor (RLR) helicase RIG-I (Steps 2, 3, and 4). The activated RIG-I molecule binds to MAVS *via* caspase activation and recruitment domain (CARD)-CARD interaction at the N-terminal domain of MAVS leading to the formation of the “MAVS Signalosome” (Step 5). Two different signals can emanate from this signalosome, the first one leads to the activation of interferon regulatory factor 3/7 (IRF3/7), transcriptional activation of Type I Interferons and stimulation of interferon stimulated genes (ISGs; Steps 6 and 7) leading to a protective antiviral immunity. The second signal phosphorylates IκB *via* IKK and releases NFκB that translocates to the nucleus and results in the transcriptional activation of pro-inflammatory cytokines (Steps 8, 9, and 10). Events at this step needs to be finely tuned as hyperstimulation of the responses may lead to a “cytokine storm” that aids in viral persistence within the infected cells. To fine tune the hyperstimualtion of the MAVS-signalosome NLRX1 antagonizes IFN signaling by binding to MAVS that may act as a brake on the hyperactive immune response. COX 5B suppress ROS production *via* suppressing MAVS aggregation and the proteasomal subunit PSMA-7 inhibits MAVS by enhancing its proteasomal degradation. Further, PCBP1, 2 and Smurf2 similarly degrade MAVS *via* K48 linked polyubiquitination and inhibit Type I Interferon production (Step 11).

Since mitochondria provide the first line of defense against viral infection, signals converging at the mitochondria need to be tightly regulated to prevent bystander tissue damage within the host. One such checkpoint is provided by the NLR, NLRX1 which prevents overactivation of the immune response by its direct competition with RIG-I at its MAVS binding site and antagonizing Type I Interferon responses (Allen et al., 2012; Qin et al., 2017). Further, ubiquitination plays an important immunomodulatory role in the MAVS-signalosome (Gack et al., 2007, 2009). The ubiquitin ligase, tripartite motif containing-25 (TRIM-25), mediates Lys63 polyubiquitination of RIG-I thus, enabling its binding with MAVS for antiviral signaling. It has been shown that TRIM-25 also ubiquitinates MAVS at Lys7 and Lys10 inducing its proteolysis and dissociating it from RIG-I to halt the antiviral signaling cascade (Castanier et al., 2012). Mitophagy induction by reactive oxygen species is another strategy for MAVS degradation at the damaged mitochondria which is sometime adopted by certain viruses to dampen the host immune response (Zhang et al., 2018; He et al., 2019). These observations suggest that stimulators of the MAVS signalosome must work in concert with the negative regulators to strike a balance between activation and deactivation in a timely manner and have been summarized in Figure 1. However, extensive studies are required to find candidate molecules that may act to dampen the overzealous immune activation following an initial protective response *via* mitochondrial sensing (D’Elia et al., 2013; Cusabio, 2020).

## Insight Into Regulation of MAVS Signalosome by RNA Viruses

### Ebola Virus

The EBOV that causes Ebola Virus Disease (EVD) is an emerging pathogen and has an almost 90% mortality rate. Although it is mostly endemic to West Africa with the Democratic Republic of Congo (DRC) being the hardest hit region over the past decade (2013–2019), it remains a major health concern worldwide due to its high potential to infect other species and the unavailability of a viable therapy till date (Kaner and Schaack, 2016; Chowell et al., 2019; Ebola virus disease, n.d.). EBOV is a non-segmented enveloped (−) single-stranded RNA virus that initially infects the innate immune cells such as macrophages (Structure of Ebola Virus, n.d.). However, the virus has the remarkable ability to infect a wide variety of cells that enables its rapid spread to different tissues. EBOV infection is characterized by hemorrhagic fever accompanied by massive cytokine storm, cytolytic damage, vascular leakage in liver, lungs, and kidneys, and ultimately death (Yu et al., 2012; Falasca et al., 2015).

The role of the viral sensor RIG-I and the subsequent activation of the MAVS pathway in determining the outcome of EBOV infection has not been thoroughly investigated. A study on mouse adapted EBOV (MA-EBOV) infection demonstrated that IFN-dependent and independent MAVS signaling takes place in an organ specific manner, where activation of monocytes and subsequent trafficking to the spleen occurs in a MAVS-dependent manner (Green et al., 2016; Dutta et al., 2017). EBOV mitigates the host immune response by using two viral IFN-antagonists, VP24 and VP35. VP35 has been shown to suppress the IFN-pathway by antagonizing the function of interferon regulatory factor (IRF) activating kinases IKKε and TANK binding kinase-1 (TBK-1; Messaoudi et al., 2015). Further, VP35 has been shown to inhibit RIG-activation *via* binding to transiently produced dsRNA during EBOV infection, thus preventing viral sensing and also by binding to the RIG-I ATPase activator PACT (Luthra et al., 2013). In an *in vitro* study, VP24 has been shown to prevent IFN-gene expression by targeting the RIG-I/MAVS pathway. It works downstream of the RIG-I/MAVS pathway by binding karyopherin 𝛼1 and inhibiting p-STAT translocation (Reid et al., 2006). Further studies are required to understand how EBOV suppress early innate immune sensing to develop antiviral strategies.

### Zika Virus

Zika virus (ZIKV) is a re-emerging mosquito borne pathogen belonging to the genus *Flavivirus*. However, apart from mosquito-transmission, several other modes of ZIKV infection have been reported and the most striking is the mother to fetus transmission *via* the transplacental route (Plourde and Bloch, 2016). Although the first document of human infection by ZIKV occurred in 1954 and was associated with mild flu-like symptoms, a recent epidemic in French Polynesia during 2013–2014 that subsequently spread to South and Central America caught the world’s attention with rising symptoms of microcephaly in newborns (Song et al., 2017; Haby et al., 2018). The re-emergence and the rising cases of ZIKV infection with higher infectivity are poorly understood and no clinically approved drug or vaccine is available till date.

ZIKV is a non-segmented enveloped (+) single stranded RNA virus that has shown to co-evolve with the host and strongly antagonizes the host antiviral IFN-responses. Several non-structural proteins of ZIKV like NS1, NS2B/3, NS4A, NS4B, and NS5 have been shown to antagonize IFN-responses (Wu et al., 2017; Ding et al., 2018; Zheng et al., 2018; Lundberg et al., 2019; Zhao et al., 2019). Studies have shown a direct interaction of the ZIKV NS4 with the N-terminal CARD domain of MAVS at the mitochondria (Ma et al., 2018). This prevents binding of RIG-I to MAVS and downstream activation of the interferon responses. It has been further shown that ZIKV NS4 specifically inhibits RIG-I mediated interferon responses and not that mediated by TLRs (Ma et al., 2018; Hu et al., 2019; Schilling et al., 2020). It is also known to disrupt mitochondrial dynamics which aids in infection (Keystone Symposia, n.d.). Further, the non-structural protein NS3 have been shown to target MAVs to proteasomal degradation *via* K48 linked polyubiquitination and subsequent downregulation of IFNβ pathway (Li et al., 2019). Further, these responses vary among the different ZIKV strains isolated from different geographical locations. ZIKV strains from Brazil and Uganda showed delayed activation of the innate immune responses mediated by RIG-I as compared to the milder Cambodia strain that correlates with their pathological outcomes (Esser-Nobis et al., 2019). Studies using these different strains in lung A549 cells revealed the important role of RIG-I sensing in early innate immune response and induction of Type I Interferon responses (Strottmann et al., 2019). However, the role of this pathway in the host tropism of different isolates of ZIKV is yet to be fully uncovered that may provide a deeper insight into the importance of early viral sensing and productive IFN-response *via* the MAVS signalosome in ZIKV clearance.

### Influenza A Virus

Influenza A virus (IAV) is one of the four types of influenza virus and the only influenza virus sub-type that has been known to cause global pandemic. Based on the presence of two surface proteins, hemagglutinin (HA) and neuraminidase (N), IAV can be sub-categorized into different strains (Bouvier and Palese, 2008). The 1918 Spanish Flu and the pandemic of 2009 were associated with the H1N1 subtype of IAV. H1N1 mostly affect children and young and middle-aged adult contrary to other flu where it affects mostly the older people.

The Influenza virus contains eight segmented (−) single stranded RNA and affects the upper respiratory tract epithelial cells causing the “seasonal” flu or fatal pulmonary disorder in extreme conditions (Shao et al., 2017). The polymeric basic 2 (PB2) subunit of the RNA polymerase complex is a major pathogenic determinant of seasonal IAV (Liu et al., 2019). Further, an intact mitochondrial membrane potential (MMP) is required for MAVS-mediated interferon production and PB2 might indirectly affect MAVS function by altering MMP (Varga et al., 2012). PB2 protein of pdm/09 variant of IAV carrying T5881 mutation has been shown to suppress MAVS-mediated interferon signaling more robustly that could potentially contribute to its increased pathogenicity. It has been demonstrated that PB2 is imported into the mitochondrial matrix and associates with MAVS at the mitochondria that correlates with reduced IFNβ production *in vitro* (Graef et al., 2010; Long and Fodor, 2016). Besides PB2, other proteins of IAV like the non-structural protein 1 (NS1) can block the RIG-I mediated induction of IFNβ by inhibiting TRIM25 (Gack et al., 2009). Inflammasome formation, a complex of NLRP3/ASC/Caspase-1, that is required for the production the inflammatory cytokine IL1β, is triggered by IFNβ in a positive feedback loop in primary lung epithelial cells and was shown to be mediated by RIG-I *via* its interaction with MAVS/TRIM2/Riplet (Mibayashi et al., 2007; Pothlichet et al., 2013). TRIM25 and Riplet positively regulates the antiviral responses mediated by RIG-I and the NS1 protein of highly pathogenic 1918 virus binds to RIG-I and TRIM25 to antagonize IFNβ activation (Gack et al., 2009; Koliopoulos et al., 2018). This correlated with the reduced induction of both Type I Interferon, as well as IL1β production by NS1 in IAV infected ferrets. Further RNAse L, a ubiquitous endonuclease for single stranded RNA, enhances NLRP3 activation and complex formation with the DExD/H-box helicase, DHX33, and MAVS in bone marrow derived dendritic cells and THP-1 derived macrophages (Chakrabarti et al., 2015). However, this antiviral response appears to be a double-edged sword as heightened inflammation and production of pro-inflammatory cytokines is often associated with increased morbidity following IAV infection. One of the NLRs, NLRX1 was shown to inhibit the production of antiviral cytokines and reduce lung pathology in IAV virus infected mice *via* its direct interaction with the RIG-I/MAVS pathway (Allen et al., 2011). Thus, NLRX1 at the mitochondria could provide a brake on the cytokine storm induced by IAV that has often been associated with the higher mortality rates during influenza virus pandemic.

### SARS-Coronavirus

Coronavirus is emerging pathogens that has serious life-threatening impact on human health. Severe acute respiratory syndrome-related coronavirus (SARS-CoV1) caused a major outbreak of respiratory disease in 2002–2004 (SARS | Home | Severe Acute Respiratory Syndrome | SARS-CoV Disease | CDC, n.d.). The current pandemic which has bypassed the death rate of all previous pandemic of the last century is caused by a novel subtype of SARS-CoV and has been named SARS-CoV2 causing corona virus disease 19 (COVID-19). Coming from zoonotic reservoir, SARS-CoV shows extreme adaptivity through species jump and is a major health concern worldwide with the probability of new strains with heightened pathogenic potential emerging every year. SARS-CoV is an enveloped (+) single stranded RNA virus and in human host, it mainly infects the ciliated epithelium and alveolar type II pneumocytes (de Wit et al., 2016). Being mostly asymptomatic in the early stages of infection with flu like symptoms, it can quickly escalate to acute respiratory distress syndrome (ARDS) and multiorgan failure (Cameron et al., 2008; Yin and Wunderink, 2018). A similar manifestation has also been observed in COVID-19 patients, where cytokine storm has been shown to result in ARDS-like symptoms. SARS-CoV-2 can efficiently alter the cytokine profile by promoting the production of pro-inflammatory genes and blocking the stimulation of interferon genes based on their mode of infectivity (i.e., severe or non-severe form of SARS; Mahmudpour et al., 2020; Ratajczak and Kucia, 2020; Wang et al., 2020a; Yap et al., 2020). An arsenal of viral proteins is dedicated for this process and the host mitochondria play a pivotal role in the early response to infection (Maier et al., 2015). SARS-CoV-1 proteins, ORF-3b and nsp-10, show direct mitochondrial association where ORF-3b co-localizes with mitochondria specific markers and nsp-10 specifically interacts with NADH 4 L subunit and cytochrome c oxidase that affects mitochondrial function (Li et al., 2005; Yuan et al., 2006). It can also inhibit the MAVS downstream signaling by directly binding to STAT1 and inhibiting the TBK1/IKK𝛆 signaling. Further, the SARS-CoV-1 envelope protein has been shown to activate inflammasome formation and stimulate the production of pro-inflammatory cytokines like IL6 and TNF which makes it an attractive target for future studies (Nieto-Torres et al., 2014). Hence, studies on SARS-CoV-1 points toward the relevance of mitochondria mediated innate immune signaling pathway that may be further extrapolated to SARS-CoV-2 infection (Singh et al., 2020). No study has reported the role of the mitochondrial innate immune sensing in COVID-19 pathogenesis that may provide effective strategies to limit viral replication within the host and the generation of a protective adaptive immune response.

## Conclusion

RNA viruses have become important etiological agents of emerging pathogens in humans constituting a major percentage of all human emerging diseases including those induced by bacteria or parasites. The past decade has seen several cases of pandemics arising due to RNA viruses originating from wild life reservoirs like the Ebola, H1N1 influenza, SARS, and MERS and the recent COVID-19 pandemic. The RNA polymerases of these viruses often lack proofreading activity increasing their mutation rates during the replicative stage of the virus. This comes as a severe challenge in developing vaccine strategies and it is important to understand conserved host immune responses which may help combat a wide range of these RNA viruses.

The innate immune response, which provides the first line of defense against these RNA viruses *via* the production of Type I Interferon, is often targeted by the viruses for the successful establishment of an infection. However, priming of IFN-responses prior to an infection can be a double edged sword as cytokine storm following hyper-stimulation of the immune responses and the over production of pro-inflammatory cytokines have been shown to be associated with diseases like Ebola, Influenza, and COVID-19 (D’Elia et al., 2013; Infection-cusabio and Topics, 2020) and the mitochondria may act as a central hub in modulating these responses. MAVS dependent pathway at the mitochondria act as a critical factor for limiting virus infection and a detailed understanding of its regulation can help fine tune the host immune responses toward a productive antiviral strategy. Several molecules like NLRX1 and DUBs regulate RIG-I binding to MAVS at the mitochondria or directly target MAVS for degradation, thus acting as a counterbalance to prevent overproduction of Type I Interferons during a persistent viral infection. Further, development of agonists for the RIG-I/MAVS pathways can be used synergistically with antiviral compounds to restrict the replication of viruses at the initial stage and offer prophylactic solution to prevent such deadly outbreaks and rapid spread of RNA-virus induced infection.

## Author Contributions

PM conceived the work. SD, ND, and PM wrote the manuscript. PM prepared the figures and revised the entire manuscript. All authors contributed to the article and approved the submitted version.

### Conflict of Interest

The authors declare that the research was conducted in the absence of any commercial or financial relationships that could be construed as a potential conflict of interest.
